# Pedal edema and jugular venous pressure for volume overload in peritoneal dialysis patients

**DOI:** 10.1186/s40697-016-0091-z

**Published:** 2016-01-13

**Authors:** Michael A. Garfinkle, James Barton

**Affiliations:** Department of Medicine, University of Calgary, Calgary, AB Canada; Department of Medicine, University of Saskatchewan, Saskatoon, SK Canada

**Keywords:** JVP, Jugular venous pressure, Edema, Volume overload, Physical exam, Peritoneal dialysis

## Abstract

**Background:**

The diagnostic strength of the jugular venous pressure (JVP) and pedal edema as physical examination tools for the assessment of volume status has been minimally studied.

**Methods:**

We conducted a prospective observational study in an outpatient peritoneal dialysis clinic in Saskatoon, Canada. Patients were adult (age 18 or older) peritoneal dialysis outpatients without any history of cardiac dysfunction, a central line, and current arteriovenous fistula. JVP was assessed by both a resident and a staff nephrologist, while the presence of edema was assessed by the resident only. Likelihood ratios were calculated for the absence or presence of pedal edema as well as the JVP at multiple cutoffs. The criterion standard for volume overload was defined as an overhydration to extracellular water ratio of greater than or equal to 7 % as determined by bioimpedance (Body Composition Monitor—Fresnius Medical Care).

**Results:**

Twenty-five separate patient encounters were assessed. Twelve patients were found to be volume overloaded while 13 were euvolemic. The presence and absence of edema were both significant signs for the presence (+likelihood ratio (LR) 16, 95 % confidence interval (CI) 1.02–260) or absence (−LR 0.44, 95 % CI 0.23–0.83) of volume overload, respectively. The JVP failed to reach statistical significance for the presence or absence of volume overload at any height above the sternal angle, although precision was poor for the positive likelihood ratio at cutoffs above 3 cm and the negative likelihood ratio at the 0 cm cutoff.

**Conclusions:**

The presence of pedal edema is a good indicator of volume overload in peritoneal dialysis patients without cardiac dysfunction, although its absence cannot definitively rule out significant water excess. A JVP of 1 to 3 cm was found to be not a clinically significant sign. We are unable to comment on the diagnostic strength of a low (0 cm) or high (JVP >3 cm) due to poor precision.

## RÉSUMÉ

**Mise en contexte:**

Le test de pression veineuse jugulaire (PVJ) et la constatation physique de la présence d’œdème au niveau de la cheville sont deux outils très utilisés lors de l’examen physique des patients afin d’évaluer leur statut volumique. Toutefois, l’acuité de ces outils au plan diagnostique n’a que très peu été étudiée jusqu’à maintenant.

**Méthode:**

L’étude d’observation prospectative a été effectuée au sein d’une clinique ambulatoire de dialyse péritonéale à Saskatoon, au Canada. Les patients sélectionnés pour participer à cette étude étaient des adultes fréquentant la clinique ambulatoire de dialyse péritonéale dont les antécédents médicaux ne faisaient aucune mention de dysfonction cardiaque, de la présence d’un cathéter central au niveau de la jugulaire ni d’une fistule artérioveineuse. Les mesures de PVJ ont d’abord été effectuées par un résident en néphrologie puis contre-vérifiées par un néphrologue. Quant à la présence d’œdème au niveau de la cheville, seule l’intervention du résident a été nécessaire. Des quotients de vraisemblance (QV) ont été calculés pour établir différentes valeurs-seuil déterminant la présence ou non d’œdème au niveau de la cheville ainsi que de pression dans la veine jugulaire. Le critère principal pour définir une surcharge volumique était un ratio d’hyperhydratation extracellulaire supérieur ou égal à 7 %, tel que déterminé à l’aide d’un analyseur de composition corporelle par bio-impédance (« Body Composition Monitor » ou BCM, de la compagnie « Fresnius Medical Care »).

**Résultats:**

Des rencontres individuelles ont eu lieu avec vingt-cinq patients. Du nombre, douze étaient en situation de surcharge volumique alors que treize étaient euvolémiques. La présence d’œdème au niveau de la cheville a été un signe clinique concluant pour diagnostiquer un problème de surcharge liquidienne (+QV 16, 95 % IC : 1.02 – 260) de même que l’absence d’œdème a chaque fois traduit un état euvolémique (−QV 0.44, 95 % IC : 0.23 – 0.83). La PVJ n’a pas été jugée statistiquement significative pour évaluer le statut volumique des patients et ce, peu importe la hauteur mesurée au-dessus de l’angle sternal. De plus, un QV positif pour une mesure au-dessus de 3 cm ou des valeurs de QV négatives pour des mesures au-dessous de zéro centimètre manquent de précision.

**Conclusions:**

La présence d’œdème au niveau de la cheville s’avère un bon indicateur de surcharge liquidienne chez les patients étudiés. Par contre, l’absence d’œdème ne saurait être un critère suffisant pour écarter l’hyperhydratation. Une mesure de PVJ entre 1 et 3 cm n’a pas été retenue comme indice significatif de la surcharge volumique. Enfin, il n’a pas été possible de discuter de la force diagnostique d’une faible PVJ (0 cm) ni d’une PVJ de plus de 3 cm en raison d’un manque de précision dans la prise des mesures.

## What was known before

There are no previous studies evaluating the strength of pedal edema or jugular venous pressure for volume assessment in the peritoneal dialysis population.

## What this adds

The presence of pedal edema was found to be an excellent sign for volume excess while its absence cannot rule out hypervolemia. A jugular venous pressure (JVP) between 1 and 3 cm was found to be a sign without clinical significance, while the diagnostic significance of a very low (0 cm) or very high JVP (>3 cm) remains unknown.

## Background

While research on physical exam findings for body water volume depletion exists [1], the diagnostic strength of commonly taught exam findings such as edema and an elevated JVP for body water volume overload, independent of heart failure, has been minimally studied. This may be due to the fact that, until recently, the only adequate criterion standard for volume overload was time-consuming radio-labelled tracer assays. However, with the advent of body composition monitoring (BCM), the objective quantitative measurement of body water at the bedside has become possible. In the present study, we compared the accuracy of leg edema and the JVP as markers of volume overload in an outpatient, peritoneal dialysis (PD) population without a history of cardiac dysfunction with BCM acting as our criterion standard.

## Methods

### Ethics, consent, and permissions

We acquired the appropriate approval from the University of Saskatchewan Research Ethics Board. Written informed consent was obtained from all patients meeting the study criteria.

### Patients

We conducted a prospective study on patients presenting to our city’s outpatient peritoneal dialysis unit from November 2014 to February 2015. All subjects during any given clinic date were screened for inclusion in the study; however, not all clinic dates during the study period were used due to variations in resident availability. Subjects were considered for inclusion in our study if they were 18 years of age or older. Subjects were then excluded if they had any evidence of cardiac dysfunction including history of abnormal echocardiogram (excepting mild valvular stenosis or regurgitation), atrial fibrillation, pulmonary hypertension, coronary artery disease, or past imaging suggestive of pulmonary edema. Patients were also excluded if they had an internal jugular central line on the right side that might interfere with JVP assessment. Finally, individuals with a patent arteriovenous fistula were excluded as fistulas have been found to independently elevate central venous pressure [2]. Some, but not all, subjects were previously known to the examiners.

Baseline characteristics were recorded from all consenting patients. These included demographic data, current use of hypertensive medications, sitting blood pressure, residual GFR from the most recent 24-h urine sample, most recently measured height, weight on presentation, and volume of PD fluid present during weighing.

### Physical examination

The assessment of edema was performed by the same examiner, a senior internal medicine resident, for all patients, while the JVP was assessed independently by both the resident and the staff nephrologist responsible for the PD clinic that day. With the subject lying supine, the presence or absence of pedal edema was assessed by applying pressure just above the ankle of one arbitrarily chosen leg and observing for any skin pitting. The JVP was then examined by raising the head of the subject’s bed to between 30° and 45°. The subject was asked to turn his head to the left, and the right neck was observed for the classic biphasic waveform characteristic suggestive of the jugular vein. If the pulsation could not be identified, the head of the bed was either raised or lowered until the JVP became visible. The external or internal jugular venous pulsation, whichever was most evident, was used as both have been shown to be equally accurate [3]. For the resident examination, once the waveform was identified, the maximum height of the pulsation above the sternal angle was measured utilizing two perpendicular rulers recorded to the nearest 0.5 cm. For the staff examination, the JVP distance above the sternal angle was more roughly estimated, as it is often done in clinical practice. If the staff had written a range, the mean was taken (i.e., 2–3 cm was converted into 2.5 cm). At the time of physical examination, neither examiner was aware of the BCM results.

### Body composition monitor

After the physical examination, quantitative body water measurements were obtained via the BCM (Fresnius Medical Care). Bioimpedance measures the conductance of a small alternating current, at various frequencies, as it travels through the body. As current conducts differently with hydration status, and different frequencies conduct preferentially through either the intra- or extracellular space, the volume content of these spaces may be inferred. Overhydration may then be determined by subtracting the calculated volume from what is expected. A complete description of the calculations involved is described elsewhere [4].

With the subject lying completely flat, the two pairs of electrodes were placed on the wrist and foot of the same side and, per the manufactures guidelines, at least 2 min were allowed to pass. The patients weight (corrected by subtracting that attributable to peritoneal fluid), sex, and height were then entered and measurements taken.

Volume overload corrected for body size was defined as an overhydration (OH) to extracellular water (ECW) ratio of greater than or equal to 7 %. This value was chosen as it represents the 90th percentile in a healthy adult population [5].

### Statistical analysis

Sensitivity, specificity, and positive and negative likelihood ratios (LRs) were calculated from the raw data utilizing the BCM OH/ECW measurement as the criterion standard for volume overload. Confidence intervals for positive and negative likelihood ratios were calculated with the method described by Simel et al. [6]. If specificity or sensitivity was 100 % for a given test (meaning one or more subgroups of the 2 × 2 table had zero patients), all cells had a value of 0.5 added to avoid a zero or infinite LR without a confidence interval [7]. Edema was considered a binary variable, while the accuracy of a raised JVP was assessed as a continuous variable in 1 cm increments, from greater than 0 to greater 6 cm above the sternal angle. The kappa value for the agreement between staff and resident JVP measurement for the >3 cm cutoff was calculated with the method described by Fleiss et al. [8].

## Results

A total of 19 patients consented to participate in the study. Six patients were seen in clinic at two time points at least 3 months apart, and these were counted as additional independent measurements, giving a total of 25 individual patient encounters. Twelve subjects (12/25: 48 %) were volume overloaded by BCM measurement, while the remaining 13 (13/25: 52 %) were found to be euvolemic. No patient was hypovolemic (OH/ECW ≤7 %). The baseline characteristics and BCM fluid measurements of these encounters are found on Table 1. One patient was a recent peritoneal dialysis start and did not have residual renal function yet calculated.

**Table 1:** Characteristics of patient encounters

	Euvolemic (*n* = 13)	Volume overloaded (*n* = 12)
Age (SD)	51 (13)	66 (11)
Male, %	62	67
Diabetes, %	15	23
Residual GFR^a^ (SD)	4.42 (2.57)	4.18 (3.68)
Systolic BP (SD)	139 (19)	138 (19)
Diastolic BP (SD)	84 (15)	77 (11)
BMI (SD)	25 (6)	31 (6)
No hypertensive medications, %	38	23
One hypertensive medication, %	15	42
More than one hypertensive medication, %	46	38
OH, L (SD)	0.2 (0.5)	2.3 (0.8)
TBW, L (SD)	38.0 (9.3)	38.4 (9.6)
ECW, L (SD)	17.0 (4.2)	19.5 (4.7)
ICW, L (SD)	21.0 (5.5)	18.8 (5.1)
OH/ECW, % (SD)	1.0 (3.3)	11.6 (3.8)

^a^One patient in the volume-overload group did not have a residual GFR calculated

*Abbreviations*: *BP* blood pressure (sitting), *ECW* extracellular water, *BMI* body mass index, *GFR* glomerular filtration rate, *ICW* intracellular water, *OH* overhydration, *TBW* total body water

The diagnostic significance of edema and a raised JVP for volume overload are found on Table 2. The resident was unable to find the JVP in one patient in the volume-overloaded group, whereas the staff nephrologist was unable to assess the JVP in two patients in the euvolemic group. The patient totals for these exam findings were adjusted as a result. There was no agreement between the staff and resident determined JVP for the >3 cm cutoff (kappa 0.076, 95 % confidence interval (CI) −0.234–0.065). As can be seen in Table 2, the JVP as measured by both the resident and staff nephrologist was a poor diagnostic tool. At any cutoff, a low JVP failed to reach statistical significance and decrease the probability of volume overload, although poor precision excludes us from making any comments on the utility of a 0-cm JVP. No statistical significance was found for an elevated JVP at any cutoff as well, although poor precision prevents us from making any strong conclusions as to its actual diagnostic strength for values >3 cm. Conversely, the presence and absence of edema were both significant signs for the presence (+LR 16, 95 % CI 1.02–260) and absence (−LR 0.44, 95 % CI 0.23–0.83) of volume overload, respectively. We also analyzed if any additional diagnostic strength could be obtained by combining the presence of an elevated JVP (resident determined) or pedal edema as a positive test; however, strength was actually diminished for all JVP cutoffs (data not shown).

**Table 2:** Diagnostic strength of edema and JVP for volume overload

	TP	TN	FP	FN	Sensitivity (%)	Specificity (%)	Positive LR [95 % CI]	Negative LR [95 % CI]
Resident								
Edema	7	13	0	5	58	100	16 [1.02, 260]	0.44 [0.23, 0.83]
JVP >0	9	7	6	2	82	54	1.8 [0.93, 3.4]	0.34 [0.087, 1.3]
JVP >1	6	7	6	5	55	54	1.2 [0.53, 2.6]	0.84 [0.37, 1.9]
JVP >2	4	8	5	7	36	62	0.95 [0.33, 2.7]	1.0 [0.56, 1.9]
JVP >3	4	11	2	7	36	85	2.4 [0.53, 11]	0.75 [0.45, 1.2]
JVP >4	2	13	0	9	18	100	5.8 [0.31, 110]	0.82 [0.61, 1.1]
JVP >5	2	13	0	9	18	100	5.8 [0.31, 110]	0.82 [0.61, 1.1]
JVP >6	1	13	0	10	9	100	3.5 [0.16, 78]	0.91 [0.72, 1.1]
Staff								
JVP >0	12	2	9	0	100	18	1.2 [0.89, 1.7]	0.18 [0.0098, 3.5]
JVP >1	9	5	6	3	75	45	1.4 [0.73, 2.6]	0.55 [0.17, 1.8]
JVP >2	7	8	3	5	58	73	2.1 [0.73, 6.3]	0.57 [0.27, 1.2]
JVP >3	1	11	0	11	8	100	2.8 [0.12, 62]	0.92 [0.73, 1.2]
JVP >4	0	11	0	12	0	100	0.92 [0.20, 43]	1.0 [0.85, 1.2]
JVP >5	0	11	0	12	0	100	0.92 [0.20, 43]	1.0 [0.85, 1.2]
JVP >6	0	11	0	12	0	100	0.92 [0.20, 43]	1.0 [0.85, 1.2]

*Abbreviations*: *FN* false negatives, *FP* false positives, *TN* true negatives, *TP* true positives, *JVP* jugular venous pressure in cm above the sternal angle, *LR* likelihood ratio

## Discussion

We report on the diagnostic strength of pedal edema and JVP for volume assessment, using BCM as the criterion standard, in an outpatient peritoneal dialysis population without evidence of cardiac disease. For the prediction of euvolemia, the absence of edema (−LR 0.44, 95 % CI 0.23–0.83) was a better sign than a reduced JVP, except possibly when the JVP was 0 cm above the sternal angle (excellent average staff negative LR but poor precision). However, the negative likelihood ratio for edema is modest and does not reduce the probability of volume overload by much. For example, given the prevalence of volume overload in our population of 48 %, the absence of pedal edema results in a post-test probability of 27 %, which is still quite a significant proportion.

As for the prediction of volume overload, no conclusions can be drawn for the diagnostic strength of an elevated (>3 cm) JVP due to our study’s small sample size and resultant wide confidence intervals. On the other hand, statistical significance was reached for the presence of edema to rule in volume overload (+LR 16, 95 % CI 1.02–260), and while the precision is also poor, it is probably an excellent sign with an average LR of over 10. In addition, there was no added diagnostic benefit of adding the JVP to the assessment for pedal edema as a tool to assess overall volume status. As no previous studies document the sensitivity and specificity of these physical exam findings, the available evidence suggests that the presence of edema is a more useful sign than an elevated JVP for volume overload.

Our 48 % prevalence of volume overload is similar to the previously reported value of 53 % by Van Biesen et al. in their large European PD bioimpedance study [9]. We were unable to find any previous studies that assessed the power of the JVP as a clinical tool to assess volume status. Review of the literature found that the central venous pressure (CVP), for which JVP is a physical exam marker, has been studied, but only in an intensive care unit population. A recent systematic review of these studies found poor correlation between CVP and measured blood volume (*r* = 0.2) [10]. Unlike our analysis, these studies did not exclude patients with cardiac dysfunction, which would be expected to interfere with the purported association between intravascular volume and central venous pressure.

Agarwal et al. evaluated the association between pedal edema and volume status in dialysis patients [11]. Contrary to our results, these authors found no association between edema and their criterion standards for volume overload. However, differing from our study, they used inferior vena cava diameter, blood volume monitoring, and brain natriuretic peptide (BNP) as their standards, which would all be expected to be less accurate than better validated measurements of body water, such as BCM. Indeed, Imaz et al. found only moderate correlation between BNP (*r* = 0.6) and inferior vena cava diameter (*r* = 0.5) with BCM in their study of stage 3 or greater CKD patients not undergoing dialysis [12].

Patients who were volume overloaded tended to be older and have lower mean diastolic pressures and a higher BMI. The higher BMI likely reflects the added weight from volume excess. We are unable to explain the increased incidence of volume overload with age, especially since both groups seemed to have similar residual renal function. The reason behind the observed lower diastolic pressure in the volume-overloaded group is equally obscure, although this could just be a chance occurrence.

Our study has several limitations. As stated, the sample size is small, limiting the precision of some of our findings. This was partly due to the fact that we had to exclude nearly half of potential subjects due to co-existing heart disease. Second, we are unable to entirely rule out the co-existence of heart disease in the population studied as some did not have a recent echocardiogram on record. Third, examiners were not blinded to the presence or absence of edema before assessing the JVP. As the JVP is one of the more subjective physical exam findings, this could have influenced our results. Furthermore, while both the examiners were trained and experienced in the JVP technique, different individuals may have obtained more accurate results. Fourth, our results are limited to dialysis patients without heart disease. As heart disease is quite prevalent among dialysis populations, this limits the more general application of our results. Fifth, while BCM has been shown to correlate very well with traditional reference standards of body water in hemodialysis patients, there is still some variation, with one study finding an error of −0.9 ± 1.4 L for ECW when compared to bromide dilution [4]. Thus, our criterion standard is close to, but does not correlate completely with, the gold standard, which could have confounded our results. We note that were unable to study intravascular volume, as BCM is unable to measure this quantity. Sixth, only one of the examiners evaluated for the presence of pedal edema.

Finally, it can be argued that increased abdominal pressure from abdominal fluid in our patients (most of whom had an ongoing dwell during assessment) could increase the JVP independent of total volume status. However, when a prolonged abdominal pressure is applied onto healthy patients without cardiac dysfunction in multiple studies of the abdominojugular reflux, the JVP is found to only transiently elevate [13]. As we excluded patients with significant cardiac dysfunction from our study population, we believe this behavior should also apply to them. In addition, ongoing dwell may affect BCM measurements, even though it is thought to be an “invisible” body compartment with respect to the machine. Arroyo et al. studied the effect of abdominal dwell on the BCM measurements and found a small (~1 %) difference in OH/ECW values between the filled and un-filled states [14]. However, we do not believe this error is large enough to have unduly affected our results.

## Conclusions

Overall, this study suggests that the utility of historically important physical examination tools (the JVP and peripheral edema) are not as accurate as previously thought after being rigorously reviewed as only the presence of pedal edema was found to be a strong clinical sign. As such, we should consider adding more accurate and consistent evaluation methods for volume status, such as BCM, into our everyday practice as this may influence patient outcomes. While the effect of BCM on clinical outcomes in peritoneal dialysis patients is unknown, BCM measurements for volume assessment, rather than routine clinical practice, have been associated with better blood pressure control and reduced left ventricular hypertrophy [15], as well as decreased mortality [16], in hemodialysis patients.
